# Hepatocellular Carcinoma in Hepatitis B Virus-Infected Patients and the Role of Hepatitis B Surface Antigen (HBsAg)

**DOI:** 10.3390/jcm11041126

**Published:** 2022-02-21

**Authors:** Satinder P. Kaur, Arslan Talat, Hamidreza Karimi-Sari, Andrew Grees, Hao Wei Chen, Daryl T. Y. Lau, Andreea M. Catana

**Affiliations:** 1 Liver Center, Department of Medicine, Beth Israel Deaconess Medical Center, Harvard Medical School, Boston, MA 02115, USA; skaur3@bidmc.harvard.edu (S.P.K.); hchen7@bidmc.harvard.edu (H.W.C.); dlau@bidmc.harvard.edu (D.T.Y.L.); 2 Division of Gastroenterology, University of Massachusetts, Worcester, MA 01605, USA; arslantalat@gmail.com; 3 Middle East Liver Diseases Center, Tehran 1598976513, Iran; karimisari.hamid@gmail.com; 4 Stony Brook Southampton Hospital, Southampton, NY 11968, USA; andrewelias_90@yahoo.com

**Keywords:** hepatocellular carcinoma, hepatitis B, chronic, hepatitis B surface antigen

## Abstract

Hepatocellular carcinoma (HCC) is the fifth most common cancer and the second leading cause of cancer-related death worldwide. Hepatitis B virus (HBV) infection is among the main risk factors for HCC. The risk of HCC is not eliminated completely after viral suppression, due to HBV DNA integrated into human chromosomes. Cirrhosis, HBV viral DNA levels, age, male gender, the immune response of the host against HBV, and a combination of obesity and diabetes are among the main risk factors for HCC. Active viral replication and long-standing active disease with inflammation are associated with a higher risk of HCC. Treatment of HBV with nucleos(t)ide analogues (NAs) decreased HCC risk by effectively decreasing viral load and inflammation. Similar risk factors have been reported in hepatitis B patients after seroclearance. Studies have reported decreased risk of HCC after seroclearance, but there were also conflicting results from a few studies indicating no difference in risk of developing HCC. The difference in HCC rates could be because of other factors such as coinfection, occult HBV infection, family history, HBV genotype, and other comorbidities. Due to the persistent risk of HCC after seroclearance, HCC surveillance is critical for early detection, especially in high-risk patients. However, long-term studies might be needed to further validate the results.

## 1. Introduction

Globally, hepatocellular carcinoma (HCC) is the fifth most common cancer and the second leading cause of cancer-related mortality. With 854,000 newly diagnosed patients, HCC led to 810,000 deaths annually from 1990 to 2015 [1]. Hepatitis B virus (HBV), hepatitis C virus (HCV), alcohol intake, non-alcoholic steatohepatitis, and ingestion of the fungal metabolite aflatoxin B1 are the main risk factors for HCC development [2].

In the high endemic regions such as East Asia and Africa, HBV accounts for 30–50% of HCC cases [3]. HBV is a DNA virus that can integrate into host chromosomes and interact with oncogenes and tumor suppression genes. HBV-induced HCC can, therefore, occur in the absence of advanced fibrosis. The risk of HCC remains highest among those with cirrhosis [4]. HCC surveillance is an effective measure for early cancer detection, especially in high-risk patients. Regular close observation with ultrasound, with or without serum alpha-fetoprotein (AFP), is cost-effective and can increase the patients’ survival [5,6].

Active viral replication and long-standing active disease with inflammation are associated with a higher risk of HCC [7]. Prolonged HBV therapy with optimal HBV DNA suppression can reduce HCC incidence and its related mortality.

Covalently closed circular DNA (cccDNA) is an episomal minichromosome located in the host cell nucleus. The genomic relaxed circular DNA (rcDNA) enters the hepatocyte nucleus via the infectious virion nucleocapsid and converts to cccDNA. The cccDNA is a template for the transcription of viral RNAs such as pgRNA and other viral mRNAs [8,9]. Complete cure is established by undetectable HBsAg in serum and eliminated HBV DNA, intrahepatic cccDNA, and integrated HBV DNA [8]. The complete cure is difficult to achieve in chronic hepatitis B (CHB), since HBV DNA is integrated into the host genome, and viral cccDNA is detected in the liver even after a sterilizing cure, which can cause a reactivation. Therefore, the term “functional cure” was coined during the combined American Association for the Study of Liver Diseases (AASLD) and European Association for the Study of the Liver (EASL) workshop in 2016. It was defined as sustained, undetectable HBV surface antigen (HBsAg) and HBV DNA in serum with or without seroconversion to HBsAb, and selected as the goal for newer HBV therapies [8]. Despite treatment and seroclearance of HBV, the risk for HCC persists in some patients. This review will focus on HCC risk in patients with CHB after viral suppression.

## 2. Risk Factors for HCC Development in Chronic HBV Infection

Liver cirrhosis seems to be the most significant risk factor for the development of HCC during NA therapy [10]. The annual HCC incidence rate among patients with liver cirrhosis was 2.53% per year, and in chronic hepatitis patients, 0.34% per year. A similar study found that the most significant predictor of HCC is cirrhosis, followed by male gender and age [11]. Another study reported the presence of cirrhosis, male gender, increasing age, higher HBV DNA levels, and core promoter mutations as independent risks for the development of HCC (*p* < 0.005) [12,13]. Shyu et al. reported that diabetic CHB patients had a 3.3% increased risk of HCC development and a 2.8% increased risk of HCC-related mortality compared to non-diabetic CHB patients [14]. Metabolic syndrome, type 2 diabetes, and central obesity independently increased the risk of HCC development in an HBV-infected population in mainland China [15]. Viral and host factors have synergistic effects on HCC development. The immune response of the host against HBV infection that can result in cirrhosis progression is an important contributory risk factor.

Similar risk factors have been implicated in the occurrence of HCC after HBV seroclearance. Age is a major independent risk factor. Studies showed that cut-off points of ≥45 years [16,17] and ≥50 years [18,19,20] are associated with an increased risk of HCC. Male gender was also independently associated with increased risk of HCC [16,17,19]. Yip et al. demonstrated that female patients aged ≤50 years had zero risk for HCC incidence within 5 years of follow-up [19]. Sex hormones may explain the difference in the incidence of HCC based on gender [21]. Moreover, patients with cirrhosis continue to have an independently higher incidence of HCC even after HBV seroclearance [17,22]. Additionally, concurrent HCV [23] or HIV infection has been identified as one of the main risk factors for HCC development [24]. The effect of HIV and HCV coinfection is less investigated, since the coinfected patients are excluded from most studies. Recent studies also showed an increased risk of HCC after seroclearance in patients with diabetes mellitus [25]. Similarly, Chen et al. showed that a combination of obesity and diabetes increased the risk of HCC more than 100-fold in HBV or HCV carriers [26]. Song et al. [27] had similar findings with a higher body mass index (≥25.0 kg/m^2^), reporting a significant association with HCC risk in HBsAg spontaneous seroclearance participants. The fatty liver was more prevalent in HCC patients in this group compared with patients having persistent HBsAg.

## 3. Risk of HCC Development in Chronic HBV-Infected Patients Treated with Older Therapies

Interferon-alpha (IFN-α), the first treatment option for CHB introduced in the latter 1980s, could induce a progressive decline in serum HBV DNA followed by a normalization of serum transaminases (ALT) in both HBeAg-positive and -negative CHB patients [28]. IFN-α is a form of immunotherapy with known anti-tumor effects [29]. Most studies of IFN-α have shown its favorable effects on HCC risk and survival.

A meta-analysis by Yang et al. showed a reduced incidence of HCC and cirrhosis in patients treated with IFN-α compared to untreated patients (relative risk [RR]: 0.59 and 0.65, respectively, *p* < 0.05) [30]. Zonneveld et al. presented similar results with reduced risk of developing HCC and improved survival in patients treated with IFN-α [31]. They also reported decreased necroinflammation and less progressive fibrosis in these patients on histology along with an increased rate of HBsAg seroclearance. In a meta-analysis of eight studies, Miyake et al. also showed the preventive effects of IFN-α against HCC in CHB. But these results were limited to the HBeAg-positive Asian population, without any significant reduction in patients from Europe [32]. Overall higher incidence of HCC in the Asian population and other factors including HBV genotype, family history of HCC, and cirrhosis may explain this difference. After a 15-year follow-up, Lin et al. showed a reduction in HCC development among patients with CHB treated with IFN-α, even with pre-existing cirrhosis [33]. Contrarily, IFN-α did not show a beneficial effect in preventing HCC in a study by Yuen et al. but, many of their patients belonged to younger age groups. They reported that five treated patients developed HCC as compared to two untreated patients [34].

Therefore, most studies suggested favorable outcomes in terms of HCC risk with IFNα treatment, with a further effect in sustained responders. But IFN-α is associated with more frequent side effects compared to NA therapy [35]. Sung et al. included 17 studies in their meta-analysis, with 12 studies evaluating the IFN-α therapy and five studies on NA therapy (mostly with lamivudine, with some patients receiving Adefovir rescue). Studies enrolling patients treated by IFN-α vs. controls showed a 34% reduced risk, and the studies enrolling patients treated by NA vs. controls showed a 78% reduced risk of HCC after treatment [36]. These results were not surprising, as treatment with NA is more effective in lowering the HCC development risk, maybe through more potent and persistent suppression of viral replication. The decreased risk of HCC was more prominent among HBeAg-positive patients, probably due to higher viral loads.

Lamivudine was approved by the Food and Drug Administration (FDA) in 1998. Along with other NA therapies, lamivudine seems to have better virologic and histological benefits for both HBeAg-positive and negative patients [37]. Yuen et al. reported that long-term lamivudine therapy was associated with reduced risks of cirrhosis and HCC development in patients without advanced disease through suppression of viral activity. Although the development of YMDD-MT mutations reduced the maximal benefit of lamivudine, the outcome was still significantly better among these patients than in untreated patients [38]. Another retrospective study reported that the annual incidence of HCC was lower among the sustained responders to lamivudine compared to the controls (0.95% vs. 4.1%, *p* = 0.005). The yearly HCC incidence rate increased to 2.18% in patients with viral breakthrough and 5.26% among those with a suboptimal response [39]. Due to the high probability of drug resistance with long-term therapy, lamivudine is not the first-line optimal CHB treatment. Studies reported that lamivudine resistance increases the HCC risk in anti-HBe positive cirrhosis [40,41].

## 4. Risk of HCC Development in Chronic HBV-Infected Patients Treated with Newer Therapies

The AASLD guidelines recommend entecavir or tenofovir as first-line NA therapies due to their potency and high genetic barrier to resistance, particularly in NA-naïve patients [42]. Studies have shown that these newer agents decrease the risk of HCC in CHB patients.

A nationwide population-based retrospective study from Taiwan compared a cohort of 21,595 NA-treated CHB patients (entecavir, lamivudine, telbivudine, or combination therapy) with 21,595 matched untreated patients. They reported that the 7-year HCC incidence rate was lower in the NA-treated patients compared to the untreated cohort (7.3% vs. 22.7%, *p* < 0.001) after adjusting for confounders. Interestingly, this association remained in a subgroup analysis of younger, non-cirrhotic, and non-diabetic patients [43]. A Japanese cohort reported that the cumulative 5-year HCC incidence was significantly lower with entecavir therapy than with no treatment (3.7% vs. 13.7%). This effect was more notable among the patients with a higher risk for HCC development. Moreover, subgroup analysis showed that HCC risk reduction was improved in the entecavir group compared to matched lamivudine-treated patients [44]. Another Taiwanese study of entecavir-treated patients showed similar risk reduction in HCC, especially in those patients who had prior lamivudine- or adefovir-resistant mutants [45]. While many studies validated the effectiveness of entecavir in reducing HCC risk, Wong et al. also demonstrated the reduction in liver-related and all-cause mortality with this medication in CHB patients with cirrhosis [46].

This HCC risk reduction has also been seen with tenofovir therapy. In an Asian population-based retrospective study, Nguyen et al. reported that the 8-year cumulative HCC incidence was higher in the matched untreated group than in the tenofovir treated group (20.13% vs. 4.69%, *p* < 0.001) [11]. In a large European study involving 10 centers and a total of 1951 adult Caucasian CHB patients, HCC risk decreased after the first 5 years of entecavir/tenofovir therapy, particularly in those with compensated cirrhosis at baseline [47]. Interestingly, the yearly incidence rate of HCC did not differ within and after 5 years in patients without cirrhosis, but it significantly declined in patients with cirrhosis. However, Kim et al. reported that among patients without cirrhosis treated with tenofovir, the observed incidence of HCC was significantly lower than predicted [48].

## 5. Risk of HCC Development in Chronic HBV-Infected Patients Treated with Entecavir vs. Tenofovir

As discussed, both of these newer agents have shown significantly reduced risk of HCC, but the comparison between the two drugs has been a matter of debate. A South Korean study [49] used a nationwide database comparing entecavir (*n* = 11,464) with tenofovir (*n* = 12,692) between 2012 to 2014. During follow-up, HCC developed in 984 patients (590 in the entecavir vs. 394 in the tenofovir group). After multivariate-adjusted analysis, the incidence of HCC was significantly lower in the tenofovir group compared to that in the entecavir group. The data were validated in a hospital cohort of adult treatment-naive CHB patients. The HCC risk was also significantly lower in the tenofovir group and diverged between the groups after 2 years of follow-up.

Dave et al. [50] compared HCC risk between entecavir and tenofovir in a meta-analysis of 14 studies with 263,947 person-years of follow-up. The unadjusted analysis showed no difference between the groups (incidence rate ratio [IRR]: 1.28, 95% CI: 0.99–1.66). However, utilizing adjusted data from seven studies, HCC risk was 27% higher in the entecavir group than in the tenofovir group. Moreover, there was no difference between these two medications among the patients with cirrhosis. These results should be interpreted with caution due to moderate heterogeneity in these estimates.

A Korean study [51] recruited data from four academic hospitals from 2012 to 2014. They evaluated a total of 2897 CHB patients: 1484 treated with entecavir and 1413 treated with tenofovir. Among all patients, 240 (8.3%) developed HCC: 138 in the entecavir and 102 in the tenofovir group. The difference in annual HCC incidence was not statistically significant between the two groups. The propensity score-matched analysis also showed similar results regarding HCC incidence, liver transplantation, and death, which were comparable between the two groups. These results can be affected by a non-random treatment allocation and the socioeconomic status of the selected patients (which was adjusted by subgroup analysis). Additionally, the unitality of HBV genotype C and including unfavorable comorbidities such as diabetes, hypertension, obesity, and smoking can contribute to NASH and NAFLD with a higher risk of HCC development in the selected groups.

A Spanish study [52] recruited 661 patients (187 received entecavir and 424 received tenofovir). Fourteen patients developed HCC; three were on entecavir (1.6%) vs. 11 on tenofovir (2.5%). Seventy percent of the HCC patients had liver cirrhosis. HCC developed in these patients despite persistent viral suppression with undetectable viral DNA. All HCC patients were men. The revalidation of the Page-B score was >17 to benefit from the surveillance of the high risk of developing HCC. About 70% of these HCC patients had cirrhosis [52,53] or decompensated cirrhosis [53]. Only 30% developed HCC without any cirrhosis [54]. Oral antiviral drugs with a high barrier to resistance made overall and liver-related 8-year survival of CHB patients (with or without compensated cirrhosis) similar to the general population.

Papatheodoridis et al. [55] studied a cohort of 1935 Caucasian patients with CHB. They showed that long-term entecavir and tenofovir monotherapies were similar regarding the patients’ HCC risk, biochemical/virological remission rate, HBsAg loss, and liver transplantation or death. However, elastographic reversion of cirrhosis after 5 years of follow-up was more prevalent after tenofovir compared to entecavir. Another retrospective cohort study [56] in 1340 consecutive NA-naïve CHB patients reported a similar risk for HCC development after entecavir and tenofovir treatment.

To summarize, conflicting results have been reported. Most studies showed no significant difference between the two drugs regarding lowering HCC or mortality rates. It is crucial to consider other risk factors for HCC development such as obesity, diabetes, alcohol consumption, smoking, age at diagnosis, and other lifestyle modifications. Moreover, patients with high viral HBV DNA for long periods are at higher risk of HCC development [57] even with treatment. Despite NA treatment, the risk of HCC development persists even after seroclearance [58,59].

Table 1 shows the studies comparing the HCC risk in CHB patients after treatment with entecavir vs. tenofovir.

## 6. Risk of HCC Development in Chronic HBV-Infected Patients after HBV Seroclearance

HBV seroclearance, defined as two negative HBsAg assays at least 6 months apart, is a rare event. A systemic review and meta-analysis by Yeo et al. [61] included 34 studies and determined the pooled annual rate of HBsAg seroclearance to be 1.02%, which included spontaneous and treatment-induced seroclearance. Patients experienced higher rates of HBsAg seroclearance if they were HBeAg-negative at baseline or had lower baseline HBV DNA and lower HBsAg (especially < 100 IU/mL) levels.

Although HBV seroclearance is associated with a lower risk of liver-related complications such as HCC, this risk still persists after seroclearance. Ahn et al. [22] showed a significant reduction of necroinflammation on liver biopsy of patients before and after HBV seroclearance. Unfortunately, all of the patients demonstrated the presence of HBV DNA, and the fibrosis score did not change significantly (*p* = 0.072). A meta-analysis by Kuang et al. [62] showed that HCC occurred in 1.86% of patients during a follow-up of 19.6 to 336 months after HBsAg seroclearance compared to 6.56% patients who were HBsAg-positive (*p* < 0.001). However, Gounder et al. [63] showed that HBV seroclearance was not associated with reduced HCC risk compared with a matched control group (*p* = 0.65). The difference in HCC rates could be because of other factors such as coinfection, family history, HBV genotype, and other comorbidities.

The exact mechanism of HCC development after HBsAg clearance is unclear. The possible mechanisms are due to cirrhosis progression, necroinflammation caused by HBV infection, and the oncogenic effect of HBV genome integrated into the human hepatocyte chromosomes [18,19]. In a study by Choi et al., the patients’ age, male sex, and the presence of liver cirrhosis at the time of HBsAg clearance were shown to be the predictors of HCC development in both univariate and multivariable analyses. Additionally, the patients’ serum levels of total bilirubin and platelet counts were the risk factors for HCC development in univariate analysis. However, the serum levels of albumin, creatinine, and AFP, prothrombin time, the presence of diabetes, and anti-HBs seroconversion could not predict HCC development in chronic HBV-infected patients after HBsAg clearance. This study also showed that PAGE-B scores with cut-off values of low- (≤9), intermediate- (10–17), and high-risk (≥18) could predict the risk of HCC development after HBsAg clearance. The PAGE-B of ≤9 had a negative predictive value of 100% for up to 10 years of follow-up [64]. Moreover, the systematic review by Kuang et al. mentioned male gender, cirrhosis, and age ≥50 years at the time of seroclearance as predictors of HCC development [62]. Patients with at least one of the abovementioned risk factors after HBsAg clearance may need close HCC surveillance.

After seroclearance, HBV DNA may remain detectable in the serum or liver cells (occult HBV infection, OBI). Patients with OBI have an increased risk of HCC development [65,66]. In a study by Wong et al., 69% (62 out of 90) of HBsAg-negative patients with HCC had OBI. Of these, 29 patients had detectable cccDNA in their liver cells, and 43 patients had HBV DNA integrated into the DNA of their liver cells near hepatic oncogenes [66]. Another study, by Huang et al., reported HCC in 90 out of 251 patients with OBI and a focal liver lesion [67]. Although the exact mechanism of HCC development in patients with OBI is unclear, it could be due to the integrated HBV DNA in the liver cells, pre-existing necroinflammation, or produced pro-oncogenic proteins [65,66].

## 7. Risk of HCC Development after Treatment-Induced or Spontaneous HBV Seroclearance

HBV seroclearance can be achieved spontaneously or with treatment. Table 2 and Table 3 describe differences between studies with spontaneous as compared to treatment-induced seroclearance, respectively. Nam et al. studied 4061 HBsAg-positive patients retrospectively and found 47 patients to have spontaneous seroclearance. Nine of these patients developed HCC [68]. Huo et al. studied 1355 chronic carriers from 1985 to 1997 and observed spontaneous HBsAg clearance in 55 patients. During a mean follow-up of 23 months, 18 patients developed serious complications, including 11 with HCC, six with cirrhosis, and one with sub-fulminant liver failure [16].

Studies including NA treatment-induced seroclearance showed similar results. A Korean study [69] evaluated 5409 CHB patients receiving NA therapy for a median of 6.8 years. They found that HBsAg seroclearance could reduce the HCC development risk (94% reduction from treatment initiation and 87% reduction from HBsAg seroclearance, *p* < 0.05). All of these HCC patients had liver cirrhosis at baseline despite HBsAg seroclearance. In a study by Yip et al. [19] that included a large cohort of 4568 patients (17% on NA therapy), 54 patients developed HCC after 3.4 years of follow-up. Age above 50 years (*p* = 0.002) and male gender (*p* = 0.01) were two independent risk factors for HCC development. Female patients aged ≤50 years had zero risk of HCC within 5 years of follow-up. They also showed that diabetes was associated with an increased risk of HCC after adjustment for age, sex, presence of cirrhosis, and use of medications (*p* = 0.036) [25]. Yuen et al. [20] reported that the cumulative risk for HCC was higher in patients with HBsAg seroclearance at ages ≥50 years (*p* = 0.004).

Chen et al. compared spontaneous (*n* = 312) and NA treatment-induced HBV seroclearance (*n* = 110) [70]. During a mean period of 75.3 months, five patients (four in spontaneous and one in treatment group) developed HCC. There were no significant differences in HCC development, overall mortality, variceal bleeding, or incidence of anti-HBs seroconversion between the two groups. Further studies are needed to compare these groups.

Some of these results should be considered with caution. Despite having two negative HBsAg assays 6 months apart, some studies [16,22,71,72] had patients with positive serum HBV DNA in their cohort. The majority of patients with cleared HBsAg have undetectable HBV DNA in their serum. In some patients, HBV DNA persists in the serum and liver despite negative HBsAg. These patients have occult HBV infection, defined as HBV DNA presence despite the absence of HBsAg. The occult HBV infection existed in 24 (73%) cryptogenic HCC patients [73]. A study by Hosseini et al. reported that the HBsAg escape mutants were detected in more patients in the HCC/Cirrhotic group than in the asymptomatic carriers [74]. Wong et al. reported that in 90 HbsAg-negative patients with HCC, 70% had evidence of occult HBV infection, and 70% had integration of HBV DNA into liver cell DNA, while 90% of them were not cirrhotic [66]. Therefore, the integration of HBV DNA remains an important etiology for developing HCC in these patients.

The cost-effectiveness of HCC surveillance after HBV seroclearance is unknown. According to the AASLD guidelines, HCC surveillance should continue after HBV seroclearance if the patient has cirrhosis, a first-degree family member with HCC, or a long duration of infection (>40 years for males and >50 years for females who have been infected with HBV at a younger age) [75].

## 8. Conclusions

The risk of HCC development persists after viral suppression and is affected by various factors. Integrated viral DNA remains the major culprit for the development of HCC after viral suppression. The combination of viral and host factors has synergistic effects on HCC development. Moreover, host immune response to HBV infection results in progression to cirrhosis and is a major contributory risk factor for HCC. Other independent risk factors for HCC are patient gender, age, type 2 diabetes, metabolic syndrome, and core promoter mutations. Active viral replication and long-standing active disease with inflammation are associated with a higher risk of HCC. Therefore, HBV treatment with the target of reducing viral loads could reduce the incidence of HCC and its related mortality. NA therapy appears to be more effective in lowering the risk of HCC development, probably through more potent and persistent suppression of viral replication. Finally, risk stratification should be performed after viral suppression to determine the long-term HCC surveillance for the patient.

## Figures and Tables

**Table 1:** Studies comparing the hepatocellular carcinoma (HCC) risk in chronic hepatitis B (CHB) patients treated with entecavir vs. tenofovir.

First Author	Year	Design	Race	Country	Mean Age (yr)	Males ETV *n* (%)	Males TDF *n* (%)	Total Patient *n* (%)	ETV *n* (%)	TDF *n* (%)	HCC in ETV *n* (%)	HCC in TDF *n* (%)	Cirrhosis before ETV *n* (%)	Cirrhosis before TDF *n* (%)	HbeAg Pos ETV *n* (%)	HbeAg Pos TDF *n* (%)	HBV DNA Baseline ETV (Log IU/mL)	HBV DNA Baseline TDF (Log IU/mL)	BMI ETV	BMI TDF	DM ETV *n* (%)	DM TDF *n* (%)
Riveiro-Barciela [52]	2017	Cohort	Cauca-sian	Spain	50 ± 13	139 (74.3)	305 (71.9)	611	187 (31)	424 (69)	3 (1.6)	11 (2.5)	64 (34.2)	133 (31.4)	34 (18.2)	67 (15.8)	4.9 ± 2.4	3.8 ± 2.3	-	-	-	-
Seung Up Kim [51]	2019	Cohort	Asian	South Korea	48.4 ± 11.7	889 (59.9)	913 (64.6)	2897	1484 (51.2)	1413 (48.7)	138 (9.2)	102 (7.2)	499 (33.6)	411 (29.1)	758 (51.1)	694 (49.1)	5.7 ± 2.1	5.4 ± 2.1	23.8 ± 4.5	23.8 ± 2.9	121 (8.15)	106 (7.5)
Jonggi Choi [49]	2019	Cohort	Asian	Korea	48.8 ± 10.5	965 (61.9)	692 (60.6)	2701	1560 (57.7)	1141 (42.2)	115 (7.3)	39 (3.4)	935 (59.9)	653 (57.2)	853 (54.7)	641 (56.2)	6.7	6.4	_-_	_-_	94 (6)	62 (5.4)
Sung Won Lee [57]	2020	Cohort	Asian	South Korea	47	926 (58.5)	841 (58.4)	3022	1583 (52.3)	1439 (47.6)	84 (5.3)	50 (3.5)	567 (35.82)	483 (33.56)	974 (61.5)	823 (57.1)	6.49 (5.28, 7.67)	6.41 (5.34, 7.49)	23.7	23.7	159 (10)	105 (7.3)
Terry Cheuk-Fung Yip [60]	2020	Cohort	Asian	China	52.9 ± 13.2	18,094 (47.3)	591 (45.1)	29,350	28,041 (95.5)	1309 (4.5)	1386 (4.9)	8 (0.6)	3822 (13.6)	38 (2.9)	8317 (29.7)	721 (55.1)	5.3 ± 2.2	4.9 ± 2.7	-	-	6366 (22.7)	93 (7.1)
Ingyoon Ha [56]	2020	Cohort	Asian	South Korea	45	558 (61)	266 (63)	1340	921 (68.7)	419 (31.2)	82 (8.9)	24 (5.7)	259 (28)	39 (9.3)	488 (53)	261 (62)	6.36	6.67	22.2	22.3	98 (11)	22 (5)
George V. Papatheodoridis [55]	2020	Cohort	Cauca-sian	Europe	52 ± 14	538 (70)	827 (71)	1951	772 (39.5)	1163 (59.6)	50 (6.5)	93 (8)	166 (21.5)	358 (30.8)	110 (14.2)	233 (20)	-	-	26.1 ± 3.8	25.8 ± 5.3	39 (7)	77 (10.8)

Abbreviations: entecavir (ETV); tenofovir (TDF); hepatocellular carcinoma (HCC); hepatitis B e antigen (HBeAg); body mass index (BMI); diabetes mellitus (DM).

**Table 2:** Studies with spontaneous seroclearance and incidence of HCC.

Author	Year	Design	Country	Total, *n*	Spontaneous Seroclearance, *n* (%)	Male, *n* (%)	Age (yrs)	Cirrhosis at Seroclearance, *n* (%)	HCC in Seroclearance Group, *n* (%)	Mean Follow-Up after Seroclearance
De Franchis [76]	1993	Retrospective	Italy	92	10 (11)	-	-	-	0	-
Da Silva [77]	1996	Retrospective	Brazil	184	20 (11)	19 (95)	41.9	6 (30)	0	68.4
Huo [16]	1998	Prospective	Taiwan	1355	55 (4)	46 (84)	54	0	11 (20)	23
McMahon [78]	2001	Prospective	USA	1536	106 (7)	-	-	-	2 (2)	150
Chen [79]	2002	Prospective	Taiwan	218	218 (100)	172 (79)	44.8	29	3 (1)	63.4
Yuen [80]	2004	Retrospective	China	3843	92 (2)	65 (71)	48.8	-	5 (5)	126
Ahn [22]	2005	Prospective	South Korea	432	49 (10)	36 (8)	50	17 (35)	5 (10)	19.6
Nam [68]	2007	Retrospective	South Korea	4061	47 (1)	-	46.2	7	9 (19)	87.9
Fwu [81]	2009	Retrospective	Taiwan	780,864	31088 (4)	-	-	-	8 (<1)	96
Simonetti [72]	2010	Prospective	USA	1271	158 (12)	-	-	-	6 (4)	103.2
Lim [82]	2014	Retrospective	New Zealand	438	145 (33)	-	40	-	0	-
Liu [83]	2014	Prospective	Taiwan	2946	529 (8)	-	-	0	8 (2)	-
Ferreira [84]	2014	Retrospective	Brazil	548	40 (7)	22 (55)	46	0	0	-
Farzi [85]	2014	Prospective	Iran	399	43 (11)	-	-	-	0	-
Tseng [86]	2015	Retrospective	Taiwan	2121	338 (16)	242 (72)	-	-	5 (2)	-
Park [87]	2016	Prospective	Korea	1919	90 (5)	-	-	-	4 (4)	-
Gounder [63]	2016	Retrospective	USA	1346	238 (18)	152 (64)	28.8	-	4 (2)	140.4
Ari [88]	2016	Retrospective	Turkey	1427	84 (6)	51 (61)	-	-	0	-
Nguyen [89]	2017	Retrospective	USA	4737	52 (1)	-	49	-	1 (2)	32

**Table 3:** Studies with both spontaneous and treatment-induced seroclearance of HBV with incidence of HCC.

Author	Year	Design	Country	Total (*n*)	Seroclearance *n* (%)	Spontaneous *n* (%)	Treatment Induced *n* (%)	Male *n* (%)	Age (yrs)	Cirrhosis at Seroclearance *n* (%)	HCC in Seroclearance Group *n* (%)	Mean Follow-Up after Seroclearance
Fattovich [90]	1998	Retrospective	Europe	309	32 (10)	16 (50)	16 (50)	-	-	-	1 (3)	-
Arase [91]	2006	Retrospective	Japan	5055	231 (5)	156 (68)	75 (32)	-	-	-	2 (1)	-
Yuen [20]	2008	Retrospective	Hong Kong	298	298 (100)	285 (96)	13 (4)	211 (71)	-	-	7 (2)	-
Tong [92]	2009	Retrospective	USA	1200	35 (3)	21 (60)	14 (40)	-	-	-	4 (11)	-
Moucari [93]	2009	Retrospective	Europe	97	28 (29)	0	28 (100)	-	-	-	0	-
Kim, JH [17]	2011	Retrospective	South Korea	2870	96 (3)	91 (95)	5 (5)	77 (80)	46.4	24 (25)	6 (6)	-
Idilman [94]	2012	Retrospective	Turkey	183	10 (6)	0	10 (100)	-	-	-	0	-
Suzuki [95]	2012	Retrospective	Japan	615	69 (11)	0	69 (100)	-	-	-	0	-
Cho [96]	2014	Retrospective	South Korea	2392	166 (7)	149 (90)	17 (10)	-	-	-	10 (6)	-
Kim, GA [69]	2014	Prospective	South Korea	5409	110 (2)	0	110 (100)	84 (76)	42	34 (31)	2 (2)	72
Orito [10]	2015	Retrospective	Japan	602	13 (2)	0	13 (100)	-	-	-	0	-
Lauret [71]	2015	Prospective	Spain	612	78 (13)	56 (72)	22 (28)	56 (72)	51	12	1 (1)s	-
Kim, GA [18]	2015	Retrospective	South Korea	829	829 (100)	724 (87)	105 (13)	575 (69)	52.3	98 (12)	19 (2)	38.4
Nagaoka [97]	2016	Retrospective	Japan	50	50 (100)	47 (94)	3 (6)	45 (90)	41.5	-	2 (4)	210
Chen [70]	2016	Retrospective	Taiwan	422	422 (100)	312 (74)	110 (26)	322 (76)	50.4	5 (1)	5 (1)	107
Stelma [98]	2017	Prospective	The Netherlands	92	16 (17)	0	16 (100)	12 (75)	39.5	-	1 (6)	60
Chi [99]	2017	Retrospective	Multicenter	5872	54 (92)	0	54 (100)	47 (94)	48	-	0	19.2
Yip [19]	2017	Retrospective	China	73493	4568 (6)	3715 (81)	853 (19)	2874 (63)	-	839 (18)	54 (3)	-
Alawad [100]	2019	Retrospective	USA	787	65 (8)	19 (29)	46 (71)	52 (80)	49	-	0	115
Li [101]	2019	Retrospective	China	172	172 (100)	0	172 (100)	135 (78)	42.6	-	1 (1)	12
Wu [102]	2019	Retrospective	China	1276	238 (19)	0	238 (100)	152 (64)	36	-	0	37

## Data Availability

Not applicable.

## References

[1] Akinyemiju T., Abera S., Ahmed M., Alam N., Alemayohu M.A., Allen C., Al-Raddadi R., Alvis-Guzman N., Amoako Y., Artaman A. (2017). The burden of primary liver cancer and underlying etiologies from 1990 to 2015 at the global, regional, and national level: Results from the global burden of disease study 2015. JAMA Oncol..

[2] Llovet J.M., Zucman-Rossi J., Pikarsky E., Sangro B., Schwartz M., Sherman M., Gores G. (2016). Hepatocellular carcinoma. Nat. Rev. Dis. Primers.

[3] Yang J.D., Mohamed E.A., Aziz A.O.A., Shousha H.I., Hashem M.B., Nabeel M.M., Abdelmaksoud A.H., Elbaz T.M., Afihene M.Y., Duduyemi B.M. (2017). Characteristics, management, and outcomes of patients with hepatocellular carcinoma in Africa: A multicountry observational study from the Africa Liver Cancer Consortium. Lancet Gastroenterol. Hepatol..

[4] Chayanupatkul M., Omino R., Mittal S., Kramer J.R., Richardson P., Thrift A.P., El-Serag H.B., Kanwal F. (2017). Hepatocellular carcinoma in the absence of cirrhosis in patients with chronic hepatitis B virus infection. J. Hepatol..

[5] Sarasin F.P., Giostra E., Hadengue A. (1996). Cost-effectiveness of screening for detection of small hepatocellular carcinoma in western patients with Child-Pugh class A cirrhosis. Am. J. Med..

[6] Lin O., Keeffe E., Sanders G., Owens D. (2004). Cost-effectiveness of screening for hepatocellular carcinoma in patients with cirrhosis due to chronic hepatitis C. Aliment. Pharmacol. Ther..

[7] Chen C.-J., Yang H.-I., Su J., Jen C.-L., You S.-L., Lu S.-N., Huang G.-T., Iloeje U.H., Group R.-H.S. (2006). Risk of hepatocellular carcinoma across a biological gradient of serum hepatitis B virus DNA level. JAMA.

[8] Ji M., Hu K. (2017). Recent advances in the study of hepatitis B virus covalently closed circular DNA. Virol. Sin..

[9] Guo H., Xu C., Zhou T., Block T.M., Guo J.-T. (2012). Characterization of the host factors required for hepadnavirus covalently closed circular (ccc) DNA formation. PLoS ONE.

[10] Orito E., Hasebe C., Kurosaki M., Osaki Y., Joko K., Watanabe H., Kimura H., Nishijima N., Kusakabe A., Izumi N. (2015). Risk of hepatocellular carcinoma in cirrhotic hepatitis B virus patients during nucleoside/nucleotide analog therapy. Hepatol. Res..

[11] Nguyen M.H., Yang H.-I., Le A., Henry L., Nguyen N., Lee M.-H., Zhang J., Wong C., Wong C., Trinh H. (2019). Reduced incidence of hepatocellular carcinoma in cirrhotic and noncirrhotic patients with chronic hepatitis B treated with Tenofovir—A propensity Score–Matched study. J. Infect. Dis..

[12] Yuen M.-F., Tanaka Y., Shinkai N., Poon R.T., But D.Y.-K., Fong D.Y., Fung J., Wong D.K.-H., Yuen J.C.-H., Mizokami M. (2008). Risk for hepatocellular carcinoma with respect to hepatitis B virus genotypes B/C, specific mutations of enhancer II/core promoter/precore regions and HBV DNA levels. Gut.

[13] Yeung P., Wong D.K.-H., Lai C.-L., Fung J., Seto W.-K., Yuen M.-F. (2011). Association of hepatitis B virus pre-S deletions with the development of hepatocellular carcinoma in chronic hepatitis B. J. Infect. Dis..

[14] Shyu Y.C., Huang T.S., Chien C.H., Yeh C.T., Lin C.L., Chien R.N. (2019). Diabetes poses a higher risk of hepatocellular carcinoma and mortality in patients with chronic hepatitis B: A population-based cohort study. J. Viral Hepat..

[15] Tan Y., Zhang X., Zhang W., Tang L., Yang H., Yan K., Jiang L., Yang J., Li C., Yang J. (2019). The influence of metabolic syndrome on the risk of hepatocellular carcinoma in patients with chronic hepatitis B infection in mainland China. Cancer Epidemiol. Prev. Biomark..

[16] Huo T., Wu J., Lee P., Chau G., Lui W., Tsay S., Ting L., Chang F., Lee S. (1998). Sero-clearance of hepatitis B surface antigen in chronic carriers does not necessarily imply a good prognosis. Hepatology.

[17] Kim J.H., Lee Y.S., Lee H.J., Yoon E., Jung Y.K., Jong E.S., Lee B.J., Seo Y.S., Yim H.J., Yeon J.E. (2011). HBsAg seroclearance in chronic hepatitis B: Implications for hepatocellular carcinoma. J. Clin. Gastroenterol..

[18] Kim G.-A., Lee H.C., Kim M.-J., Ha Y., Park E.J., An J., Lee D., Shim J.H., Kim K.M., Lim Y.-S. (2015). Incidence of hepatocellular carcinoma after HBsAg seroclearance in chronic hepatitis B patients: A need for surveillance. J. Hepatol..

[19] Yip T.C.-F., Chan H.L.-Y., Wong V.W.-S., Tse Y.-K., Lam K.L.-Y., Wong G.L.-H. (2017). Impact of age and gender on risk of hepatocellular carcinoma after hepatitis B surface antigen seroclearance. J. Hepatol..

[20] Yuen M.F., Wong D.K.H., Fung J., Ip P., But D., Hung I., Lau K., Yuen J.C.H., Lai C.L. (2008). HBsAg Seroclearance in chronic hepatitis B in Asian patients: Replicative level and risk of hepatocellular carcinoma. Gastroenterology.

[21] Keng V.W., Largaespada D.A., Villanueva A. (2012). Why men are at higher risk for hepatocellular carcinoma?. J. Hepatol..

[22] Ahn S.H., Park Y.N., Park J.Y., Chang H.-Y., Lee J.M., Shin J.E., Han K.-H., Park C., Moon Y.M., Chon C.Y. (2005). Long-term clinical and histological outcomes in patients with spontaneous hepatitis B surface antigen seroclearance. J. Hepatol..

[23] Yang W.-T., Wu L.-W., Tseng T.-C., Chen C.-L., Yang H.-C., Su T.-H., Wang C.-C., Kuo S.F.-T., Liu C.-H., Chen P.-J. (2016). Hepatitis B surface antigen loss and hepatocellular carcinoma development in patients with dual hepatitis B and C infection. Medicine.

[24] Hu J., Liu K., Luo J. (2019). HIV–HBV and HIV–HCV coinfection and liver cancer development. HIV/AIDS-Associated Viral OncoGenesis.

[25] Yip T.C.-F., Wong V.W.-S., Chan H.L.-Y., Tse Y.-K., Kong A.P.-S., Lam K.L.-Y., Lui G.C.-Y., Wong G.L.-H. (2018). Effects of diabetes and glycemic control on risk of hepatocellular carcinoma after seroclearance of hepatitis B surface antigen. Clin. Gastroenterol. Hepatol..

[26] Chen C.L., Yang H.I., Yang W.S., Liu C.J., Chen P.J., You S.L., Wang L.Y., Sun C.A., Lu S.N., Chen D.S. (2008). Metabolic factors and risk of hepatocellular carcinoma by chronic hepatitis B/C infection: A follow-up study in Taiwan. Gastroenterology.

[27] Song C., Zhu J., Ge Z., Yu C., Tian T., Wang H., Han J., Shen H., Dai J., Lu J. (2019). Spontaneous seroclearance of hepatitis B surface antigen and risk of hepatocellular carcinoma. Clin. Gastroenterol. Hepatol..

[28] Craxì A., Di Bona D., Cammà C. (2003). Interferon-α for HBeAg-positive chronic hepatitis B. J. Hepatol..

[29] Zhuang L., Zeng X., Yang Z., Meng Z. (2013). Effect and safety of interferon for hepatocellular carcinoma: A systematic review and meta-analysis. PLoS ONE.

[30] Yang Y.F., Zhao W., Zhong Y.D., Xia H.M., Shen L., Zhang N. (2009). Interferon therapy in chronic hepatitis B reduces progression to cirrhosis and hepatocellular carcinoma: A meta-analysis. J. Viral Hepat..

[31] Van Zonneveld M., Honkoop P., Hansen B.E., Niesters H.G., Murad S.D., de Man R.A., Schalm S.W., Janssen H.L. (2004). Long-term follow-up of alpha-interferon treatment of patients with chronic hepatitis B. Hepatology.

[32] Miyake Y., Kobashi H., Yamamoto K. (2009). Meta-analysis: The effect of interferon on development of hepatocellular carcinoma in patients with chronic hepatitis B virus infection. J. Gastroenterol..

[33] Lin S.-M., Yu M.-L., Lee C.-M., Chien R.-N., Sheen I.-S., Chu C.-M., Liaw Y.-F. (2007). Interferon therapy in HBeAg positive chronic hepatitis reduces progression to cirrhosis and hepatocellular carcinoma. J. Hepatol..

[34] Yuen M.-F., Hui C.-K., Cheng C.-C., Wu C.-H., Lai Y.-P., Lai C.-L. (2001). Long-term follow-up of interferon alfa treatment in Chinese patients with chronic hepatitis B infection: The effect on hepatitis B e antigen seroconversion and the development of cirrhosis-related complications. Hepatology.

[35] Janssen H., Brouwer J., Nevens F., Sanchez-Tapias J., Craxi A., Hadziyannis S. (1993). Fatal hepatic decompensation associated with interferon alfa. European concerted action on viral hepatitis (Eurohep). Br. Med. J..

[36] Sung J., Tsoi K., Wong V., Li K., Chan H. (2008). Meta-analysis: Treatment of hepatitis B infection reduces risk of hepatocellular carcinoma. Aliment. Pharmacol. Ther..

[37] Lau D.T.-Y., Bleibel W. (2008). Current status of antiviral therapy for hepatitis B. Ther. Adv. Gastroenterol..

[38] Yuen M., Seto W.-K., Chow D., Tsui K., Wong D., Ngai V., Wong B., Fung J., Yuen J., Lai C.-L. (2007). Long-term lamivudine therapy reduces the risk of long-term complications of chronic hepatitis B infection even in patients without advanced disease. Antivir. Ther..

[39] Eun J.R., Lee H.J., Kim T.N., Lee K.S. (2010). Risk assessment for the development of hepatocellular carcinoma: According to on-treatment viral response during long-term lamivudine therapy in hepatitis B virus-related liver disease. J. Hepatol..

[40] Andreone P., Gramenzi A., Cursaro C., Biselli M., Camma C., Trevisani F., Bernardi M. (2004). High risk of hepatocellular carcinoma in anti-HBe positive liver cirrhosis patients developing lamivudine resistance. J. Viral Hepat..

[41] Kumada T., Toyoda H., Tada T., Kiriyama S., Tanikawa M., Hisanaga Y., Kanamori A., Niinomi T., Yasuda S., Andou Y. (2013). Effect of nucleos (t) ide analogue therapy on hepatocarcinogenesis in chronic hepatitis B patients: A propensity score analysis. J. Hepatol..

[42] Terrault N.A., Bzowej N.H., Chang K.M., Hwang J.P., Jonas M.M., Murad M.H. (2016). A ASLD guidelines for treatment of chronic hepatitis B. Hepatology.

[43] Wu C.-Y., Lin J.-T., Ho H.J., Su C.-W., Lee T.-Y., Wang S.-Y., Wu C., Wu J.-C. (2014). Association of nucleos (t) ide analogue therapy with reduced risk of hepatocellular carcinoma in patients with chronic hepatitis B—A nationwide cohort study. Gastroenterology.

[44] Hosaka T., Suzuki F., Kobayashi M., Seko Y., Kawamura Y., Sezaki H., Akuta N., Suzuki Y., Saitoh S., Arase Y. (2013). Long-term entecavir treatment reduces hepatocellular carcinoma incidence in patients with hepatitis B virus infection. Hepatology.

[45] Yang S.-C., Lee C.-M., Hu T.-H., Wang J.-H., Lu S.-N., Hung C.-H., Changchien C.-S., Chen C.-H. (2013). Virological response to entecavir reduces the risk of liver disease progression in nucleos (t) ide analogue-experienced HBV-infected patients with prior resistant mutants. J. Antimicrob. Chemother..

[46] Wong G.L.H., Chan H.L.Y., Mak C.W.H., Lee S.K.Y., Ip Z.M.Y., Lam A.T.H., Iu H.W.H., Leung J.M.S., Lai J.W.Y., Lo A.O.S. (2013). Entecavir treatment reduces hepatic events and deaths in chronic hepatitis B patients with liver cirrhosis. Hepatology.

[47] Papatheodoridis G.V., Idilman R., Dalekos G.N., Buti M., Chi H., van Boemmel F., Calleja J.L., Sypsa V., Goulis J., Manolakopoulos S. (2017). The risk of hepatocellular carcinoma decreases after the first 5 years of entecavir or tenofovir in Caucasians with chronic hepatitis B. Hepatology.

[48] Kim W.R., Loomba R., Berg T., Aguilar Schall R.E., Yee L.J., Dinh P.V., Flaherty J.F., Martins E.B., Therneau T.M., Jacobson I. (2015). Impact of long-term tenofovir disoproxil fumarate on incidence of hepatocellular carcinoma in patients with chronic hepatitis B. Cancer.

[49] Choi J., Kim H.J., Lee J., Cho S., Ko M.J., Lim Y.-S. (2019). Risk of hepatocellular carcinoma in patients treated with entecavir vs tenofovir for chronic hepatitis B: A Korean nationwide cohort study. JAMA Oncol..

[50] Dave S., Park S., Murad M.H., Barnard A., Prokop L., Adams L.A., Singh S., Loomba R. (2021). Comparative Effectiveness of Entecavir Versus Tenofovir for Preventing Hepatocellular Carcinoma in Patients with Chronic Hepatitis B: A Systematic Review and Meta-Analysis. Hepatology.

[51] Kim S.U., Seo Y.S., Lee H.A., Kim M.N., Lee Y.R., Lee H.W., Park J.Y., Ahn S.H., Han K.-H., Hwang S.G. (2019). A multicenter study of entecavir vs. tenofovir on prognosis of treatment-naïve chronic hepatitis B in South Korea. J. Hepatol..

[52] Riveiro-Barciela M., Tabernero D., Calleja J.L., Lens S., Manzano M.L., Rodríguez F.G., Crespo J., Piqueras B., Pascasio J.M., Comas C. (2017). Effectiveness and safety of entecavir or tenofovir in a Spanish cohort of chronic hepatitis B patients: Validation of the page-B score to predict hepatocellular carcinoma. Dig. Dis. Sci..

[53] Papatheodoridis G.V., Dalekos G.N., Yurdaydin C., Buti M., Goulis J., Arends P., Sypsa V., Manolakopoulos S., Mangia G., Gatselis N. (2015). Incidence and predictors of hepatocellular carcinoma in Caucasian chronic hepatitis B patients receiving entecavir or tenofovir. J. Hepatol..

[54] Papatheodoridis G.V., Sypsa V., Dalekos G., Yurdaydin C., van Boemmel F., Buti M., Goulis J., Calleja J.L., Chi H., Manolakopoulos S. (2018). Eight-year survival in chronic hepatitis B patients under long-term entecavir or tenofovir therapy is similar to the general population. J. Hepatol..

[55] Papatheodoridis G.V., Dalekos G.N., Idilman R., Sypsa V., Van Boemmel F., Buti M., Calleja J.L., Goulis J., Manolakopoulos S., Loglio A. (2020). Similar risk of hepatocellular carcinoma during long-term entecavir or tenofovir therapy in Caucasian patients with chronic hepatitis B. J. Hepatol..

[56] Ha I., Chung J.W., Jang E.S., Jeong S.H., Kim J.W. (2020). Comparison of the on-treatment risks for hepatocellular carcinoma between entecavir and tenofovir: A propensity score matching analysis. J. Gastroenterol. Hepatol..

[57] Lee S.W., Kwon J.H., Lee H.L., Yoo S.H., Nam H.C., Sung P.S., Nam S.W., Bae S.H., Choi J.Y., Yoon S.K. (2020). Comparison of tenofovir and entecavir on the risk of hepatocellular carcinoma and mortality in treatment-naïve patients with chronic hepatitis B in Korea: A large-scale, propensity score analysis. Gut.

[58] Arends P., Sonneveld M.J., Zoutendijk R., Carey I., Brown A., Fasano M., Mutimer D., Deterding K., Reijnders J.G., Oo Y. (2015). Entecavir treatment does not eliminate the risk of hepatocellular carcinoma in chronic hepatitis B: Limited role for risk scores in Caucasians. Gut.

[59] Kim S.S., Hwang J.C., Lim S.G., Ahn S.J., Cheong J.Y., Cho S.W. (2014). Effect of virological response to entecavir on the development of hepatocellular carcinoma in hepatitis B viral cirrhotic patients: Comparison between compensated and decompensated cirrhosis. Off. J. Am. Coll. Gastroenterol..

[60] Yip T.C., Wong V.W., Chan H.L., Tse Y.K., Lui G.C., Wong G.L. (2020). Tenofovir Is Associated with Lower Risk of Hepatocellular Carcinoma Than Entecavir in Patients with Chronic HBV Infection in China. Gastroenterology.

[61] Yeo Y.H., Ho H.J., Yang H.-I., Tseng T.-C., Hosaka T., Trinh H.N., Kwak M.-S., Park Y.M., Fung J.Y.Y., Buti M. (2019). Factors associated with rates of HBsAg seroclearance in adults with chronic HBV infection: A systematic review and meta-analysis. Gastroenterology.

[62] Kuang X.J., Jia R.R., Huo R.R., Yu J.J., Wang J.J., Xiang B.D., Li L.Q., Peng Z., Zhong J.H. (2018). Systematic review of risk factors of hepatocellular carcinoma after hepatitis B surface antigen seroclearance. J. Viral Hepat..

[63] Gounder P.P., Bulkow L.R., Snowball M., Negus S., Spradling P.R., Simons B.C., McMahon B.J. (2016). Nested case–control study: Hepatocellular carcinoma risk after hepatitis B surface antigen seroclearance. Aliment. Pharmacol. Ther..

[64] Choi J., Yoo S., Lim Y.S. (2021). Comparison of Long-Term Clinical Outcomes Between Spontaneous and Therapy-Induced HBsAg Seroclearance. Hepatology.

[65] Mak L.Y., Wong D.K., Pollicino T., Raimondo G., Hollinger F.B., Yuen M.F. (2020). Occult hepatitis B infection and hepatocellular carcinoma: Epidemiology, virology, hepatocarcinogenesis and clinical significance. J. Hepatol..

[66] Wong D.K.-H., Cheng S.C.Y., Mak L.L.-Y., To E.W.-P., Lo R.C.-L., Cheung T.-T., Seto W.-K., Fung J., Man K., Lai C.-L. (2020). Among Patients with Undetectable Hepatitis B Surface Antigen and Hepatocellular Carcinoma, a High Proportion Has Integration of HBV DNA into Hepatocyte DNA and No Cirrhosis. Clin. Gastroenterol. Hepatol..

[67] Huang Y., Li W., Hu H.T., Ruan S.M., Xian M.F., Xie X.Y., Lu M.D., Kuang M., Chen L.D., Wang W. (2022). Contrast-enhanced US diagnostic algorithm of hepatocellular carcinoma in patients with occult hepatitis B. Abdom. Radiol..

[68] Nam S.W., Jung J.J., Bae S.H., Choi J.Y., Yoon S.K., Cho S.H., Han J.Y., Han N.I., Yang J.M., Lee Y.S. (2007). Clinical outcomes of delayed clearance of serum HBsAG in patients with chronic HBV infection. Korean J. Intern. Med..

[69] Kim G.-A., Lim Y.-S., An J., Lee D., Shim J.H., Kim K.M., Lee H.C., Chung Y.-H., Lee Y.S., Suh D.J. (2014). HBsAg seroclearance after nucleoside analogue therapy in patients with chronic hepatitis B: Clinical outcomes and durability. Gut.

[70] Chen Y., Jeng W., Chien R., Chu C., Liaw Y. (2016). Clinical outcomes after spontaneous and nucleos (t) ide analogue-treated HB sAg seroclearance in chronic HBV infection. Aliment. Pharmacol. Ther..

[71] Lauret E., González-Diéguez M.L., Rodríguez M., González M., Melón S., Rodrigo L., Rodríguez M. (2015). Long-term outcome in Caucasian patients with chronic hepatitis B virus infection after HB sAg seroclearance. Liver Int..

[72] Simonetti J., Bulkow L., McMahon B.J., Homan C., Snowball M., Negus S., Williams J., Livingston S.E. (2010). Clearance of hepatitis B surface antigen and risk of hepatocellular carcinoma in a cohort chronically infected with hepatitis B virus. Hepatology.

[73] Wong D.K.H., Huang F.Y., Lai C.L., Poon R.T.P., Seto W.K., Fung J., Hung I.F.N., Yuen M.F. (2011). Occult hepatitis B infection and HBV replicative activity in patients with cryptogenic cause of hepatocellular carcinoma. Hepatology.

[74] Hosseini S.Y., Sanaei N., Fattahi M.-R., Malek-Hosseini S.A., Sarvari J. (2019). Association of HBsAg mutation patterns with hepatitis B infection outcome: Asymptomatic carriers versus HCC/cirrhotic patients. Ann. Hepatol..

[75] Terrault N.A., Lok A.S., McMahon B.J., Chang K.M., Hwang J.P., Jonas M.M., Brown Jr R.S., Bzowej N.H., Wong J.B. (2018). Update on prevention, diagnosis, and treatment of chronic hepatitis B: AASLD 2018 hepatitis B guidance. Hepatology.

[76] De Franchis R., Meucci G., Vecchi M., Tatarella M., Colombo M., Del Ninno E., Rumi M.G., Donato M.F., Ronchi G. (1993). The natural history of asymptomatic hepatitis B surface antigen carriers. Ann. Intern. Med..

[77] Da Silva L.C., Madruga C.L.D.A., Carrilho F.J., Pinho J.R.R., Saéz-Alquezar A., Santos C., Bassit L., Barreto C., Da Fonseca L.E.P., Alves V.A.F. (1996). Spontaneous hepatitis B surface antigen clearance in a long-term follow-up study of patients with chronic type B hepatitis. Lack of correlation with hepatitis C and D virus superinfection. J. Gastroenterol..

[78] McMahon B.J., Holck P., Bulkow L., Snowball M. (2001). Serologic and clinical outcomes of 1536 Alaska Natives chronically infected with hepatitis B virus. Ann. Intern. Med..

[79] Chen Y.C., Sheen I.S., Chu C.M., Liaw Y.F. (2002). Prognosis following spontaneous HBsAg seroclearance in chronic hepatitis B patients with or without concurrent infection. Gastroenterology.

[80] Yuen M.F., Wong D.K.H., Sablon E., Tse E., Ng I.O.L., Yuan H.J., Siu C.W., Sander T.J., Bourne E.J., Hall J.G. (2004). HBsAg seroclearance in chronic hepatitis B in the Chinese: Virological, histological, and clinical aspects. Hepatology.

[81] Fwu C.-W., Chien Y.-C., Kirk G.D., Nelson K.E., You S.-L., Kuo H.-S., Feinleib M., Chen C.-J. (2009). Hepatitis B virus infection and hepatocellular carcinoma among parous Taiwanese women: Nationwide cohort study. J. Natl. Cancer Inst..

[82] Lim T.H., Gane E., Moyes C., Borman B., Cunningham C. (2015). Serological and clinical outcomes of horizontally transmitted chronic hepatitis B infection in New Zealand Māori: Results from a 28-year follow-up study. Gut.

[83] Liu J., Yang H.-I., Lee M.-H., Lu S.-N., Jen C.-L., Batrla-Utermann R., Wang L.-Y., You S.-L., Hsiao C.K., Chen P.-J. (2014). Spontaneous seroclearance of hepatitis B seromarkers and subsequent risk of hepatocellular carcinoma. Gut.

[84] Ferreira S.C., Chachá S.G., Souza F.F., Teixeira A.C., Santana R.C., Villanova M.G., Zucoloto S., Ramalho L.N., Perdoná G.S., Passos A.D. (2014). Factors associated with spontaneous HBsAg clearance in chronic hepatitis B patients followed at a university hospital. Ann. Hepatol..

[85] Farzi H., Daryani N.E., Mehrnoush L., Salimi S., Alavian S.M. (2014). Prognostic values of fluctuations in serum levels of alanine transaminase in inactive carrier state of HBV infection. Hepat. Mon..

[86] Tseng T.C., Liu C.J., Chen C.L., Yang W.T., Yang H.C., Su T.H., Wang C.C., Kuo S.T., Liu C.H., Chen P.J. (2015). Higher lifetime chance of spontaneous surface antigen loss in hepatitis B carriers with genotype C infection. Aliment. Pharmacol. Ther..

[87] Park Y.M., Lee S.G. (2016). Clinical features of HBsAg seroclearance in hepatitis B virus carriers in South Korea: A retrospective longitudinal study. World J. Gastroenterol..

[88] Ari A., Çalik Ş., Tosun S., CAYMAZ S.Ö. (2016). A persistently low HBV DNA level is a predictor of spontaneous HBsAg clearance in patients with chronic hepatitis B. Turk. J. Med. Sci..

[89] Nguyen L.H., Hoang J., Nguyen N.H., Vu V.D., Wang C., Trinh H.N., Li J., Zhang J.Q., Nguyen M.H. (2016). Ethnic differences in incidence of hepatitis B surface antigen seroclearance in a real-life multicenter clinical cohort of 4737 patients with chronic hepatitis B infection. Aliment. Pharmacol. Ther..

[90] Fattovich G., Giustina G., Sanchez-Tapias J., Quero C., Mas A., Olivotto P.G., Solinas A., Almasio P., Hadziyannis S., Degos F. (1998). Delayed clearance of serum HBsAg in compensated cirrhosis B: Relation to interferon alpha therapy and disease prognosis. Am. J. Gastroenterol..

[91] Arase Y., Ikeda K., Suzuki F., Suzuki Y., Saitoh S., Kobayashi M., Akuta N., Someya T., Hosaka T., Sezaki H. (2006). Long-term outcome after hepatitis B surface antigen seroclearance in patients with chronic hepatitis B. Am. J. Med..

[92] Tong M.J., Nguyen M.O., Tong L.T., Blatt L.M. (2009). Development of hepatocellular carcinoma after seroclearance of hepatitis B surface antigen. Clin. Gastroenterol. Hepatol..

[93] Moucari R., Korevaar A., Lada O., Martinot-Peignoux M., Boyer N., Mackiewicz V., Dauvergne A., Cardoso A.C., Asselah T., Nicolas-Chanoine M.-H. (2009). High rates of HBsAg seroconversion in HBeAg-positive chronic hepatitis B patients responding to interferon: A long-term follow-up study. J. Hepatol..

[94] Idilman R., Cinar K., Seven G., Bozkus Y., Elhan A., Bozdayi M., Yurdaydin C., Bahar K. (2012). Hepatitis B surface antigen seroconversion is associated with favourable long-term clinical outcomes during lamivudine treatment in HBeAg-negative chronic hepatitis B patients. J. Viral Hepat..

[95] Suzuki F., Arase Y., Suzuki Y., Akuta N., Sezaki H., Seko Y., Kawamura Y., Hosaka T., Kobayashi M., Saito S. (2012). Long-term efficacy of interferon therapy in patients with chronic hepatitis B virus infection in Japan. J. Gastroenterol..

[96] Cho J.-Y., Paik Y.-H., Sohn W., Cho H.C., Gwak G.-Y., Choi M.S., Lee J.H., Koh K.C., Paik S.W., Yoo B.C. (2014). Patients with chronic hepatitis B treated with oral antiviral therapy retain a higher risk for HCC compared with patients with inactive stage disease. Gut.

[97] Nagaoka S., Abiru S., Komori A., Sasaki R., Bekki S., Hashimoto S., Saeki A., Yamasaki K., Migita K., Nakamura M. (2016). Hepatic flares promote rapid decline of serum hepatitis B surface antigen (HBsAg) in patients with HBsAg seroclearance: A long-term follow-up study. Hepatol. Res..

[98] Stelma F., Van Der Ree M., Jansen L., Peters M., Janssen H., Zaaijer H., Takkenberg R.B., Reesink H. (2017). HB sAg loss after peginterferon-nucleotide combination treatment in chronic hepatitis B patients: 5 years of follow-up. J. Viral Hepat..

[99] Chi H., Wong D., Peng J., Cao J., Van Hees S., Vanwolleghem T., Qi X., Chen L., Feld J.J., de Knegt R.J. (2017). Durability of response after hepatitis B surface antigen seroclearance during nucleos (t) ide analogue treatment in a multiethnic cohort of chronic hepatitis B patients: Results after treatment cessation. Clin. Infect. Dis..

[100] Alawad A.S., Auh S., Suarez D., Ghany M.G. (2020). Durability of spontaneous and treatment-related loss of hepatitis B s antigen. Clin. Gastroenterol. Hepatol..

[101] Li M.H., Yi W., Zhang L., Lu Y., Lu H.H., Shen G., Wu S.L., Hao H.X., Gao Y.J., Chang M. (2019). Predictors of sustained functional cure in hepatitis B envelope antigen-negative patients achieving hepatitis B surface antigen seroclearance with interferon-alpha–based therapy. J. Viral Hepat..

[102] Wu Y., Liu Y., Lu J., Cao Z., Jin Y., Ma L., Geng N., Ren S., Zheng Y., Shen C. (2020). Durability of interferon-induced hepatitis B surface antigen seroclearance. Clin. Gastroenterol. Hepatol..

